# Long Non-Coding TP73-AS1: A Potential Biomarker and Therapeutic Target in Cancer

**DOI:** 10.3390/ijms26083886

**Published:** 2025-04-20

**Authors:** Kejing Li, Dapeng Zhao, Xuena Liu, Qiyou Cao, Longzhu Ruan, Huiwen Lei, Xiaohua Chen, Xiaodong Jin, Qiang Li, Xiaodong Xie, Cuixia Di

**Affiliations:** 1 School of Basic Medical Sciences, Lanzhou University, Lanzhou 730000, China; 2 Bio-Medical Research Center, Institute of Modern Physics, Chinese Academy of Sciences, Lanzhou 730000, China; 3 College of Life Sciences, University of Chinese Academy of Sciences, Beijing 101408, China; 4 School of Nuclear Science and Technology, University of Chinese Academy of Sciences, Beijing 101408, China; 5 Department of Toxicology, School of Public Health, Lanzhou University, Lanzhou 730000, China; 6 Advanced Energy Science and Technology Guangdong Laboratory, Huizhou 516029, China

**Keywords:** long non-coding RNA, TP73-AS1, cancer, molecular mechanism, biomarker

## Abstract

Tumor protein 73 antisense RNA 1 (TP73-AS1), a newly discovered long non-coding RNA (lncRNA), the dysregulated expression of which is closely related to the occurrence, drug resistance, and prognosis of various cancers. Exploring the regulatory mechanism of TP73-AS1 provides a new research direction for cancer diagnosis and treatment. On this basis, we briefly review the molecular structural and dual regulatory roles of TP73-AS1 in cancer. In addition, we outline its three molecular mechanisms in cancer: binding to proteins, regulating signaling pathways, and serving as molecular sponges. Subsequently, we introduce the role of TP73-AS1 in common malignant tumors such as gastric cancer (GC), lung cancer, colorectal cancer (CRC), etc. Last, emphasis is given to the potential clinical value of TP73-AS1, especially as single nucleotide polymorphisms of this lncRNA are associated with the risk of GC and CRC. Therefore, this review highlights the significance of TP73-AS1 as a novel biomarker and therapeutic target.

## 1. Introduction

Cancer remains a leading cause of global mortality and morbidity, imposing significant burdens on individuals, families, and society at large [1]. While conventional therapeutic modalities, including surgery, radiotherapy, and chemotherapy, have demonstrated efficacy in managing early- and intermediate-stage cancers, their effectiveness in advanced-stage and refractory malignancies remains limited [2,3]. Therefore, there is an urgent need to find reliable tumor markers and therapeutic targets to improve the diagnosis and treatment of tumors.

In recent years, the key regulatory role of long non-coding RNA (lncRNA) in the occurrence and development of malignant tumors has become a major breakthrough in the field of cancer research [4,5]. A large number of studies have shown that lncRNA is not only deeply involved in the initiation and development of tumors but also closely related to tumor drug resistance, clinical treatment effects, and prognosis [6,7,8]. Among numerous lncRNAs, tumor protein 73 Antisense RNA 1 (TP73-AS1), also known as KIAA0495 and p53-dependent apoptosis regulator (PDAM), is one of the most extensively studied lncRNAs and plays an important regulatory role in a variety of malignant tumors. TP73-AS1 was first discovered in multiple myeloma (MM). During the progression from normal plasma cells to monoclonal gammopathy of undetermined significance (MGUS) and then to MM, its expression level shows a continuous downward trend [9]. Notably, the expression pattern of TP73-AS1 varies significantly across different cancer types. Studies have shown that TP73-AS1 is upregulated in the majority of solid tumors, including gastric cancer (GC) [10,11], lung cancer [12,13], colorectal cancer (CRC) [14], cervical cancer (CC) [15], and hepatocellular carcinoma (HCC) [16]. In contrast, TP73-AS1 expression is downregulated in certain cancers, such as bladder cancer [17]. The dual role of TP73-AS1 as both an oncogene and a tumor suppressor, depending on the cancer type, underscores its complexity and the need for further investigation into its regulatory mechanisms.

In this review, we summarize the latest findings on TP73-AS1 expression in human cancers, its molecular mechanisms, and clinical significance, with a particular focus on its potential as a diagnostic biomarker and therapeutic target, as well as the association between its single nucleotide polymorphisms (SNPs) and cancer susceptibility.

## 2. Overview of TP73-AS1 in Cancer

### 2.1. Structure of TP73-AS1

LncRNAs are a class of non-protein-coding transcripts exceeding 200 nucleotides in length. These RNA molecules are transcribed by RNA polymerase II but lack protein-coding capacity due to the absence of functional open reading frames (ORFs) [18]. Based on their genomic locations, lncRNAs can be classified into five main categories: intronic lncRNAs, intergenic lncRNAs, sense lncRNAs, antisense lncRNAs, and bidirectional lncRNAs (Figure S1a). Among these, lncRNAs located on the antisense strand can be further subdivided into five subtypes based on their overlap with the sense strand: three types of antisense lncRNAs (head-to-head at the 5′ end, tail-to-tail at the 3′ end, and full overlapping) and two types of bidirectional ones (nearby to head and nearby to tail) (Figure S1b).

**Figure 1:**
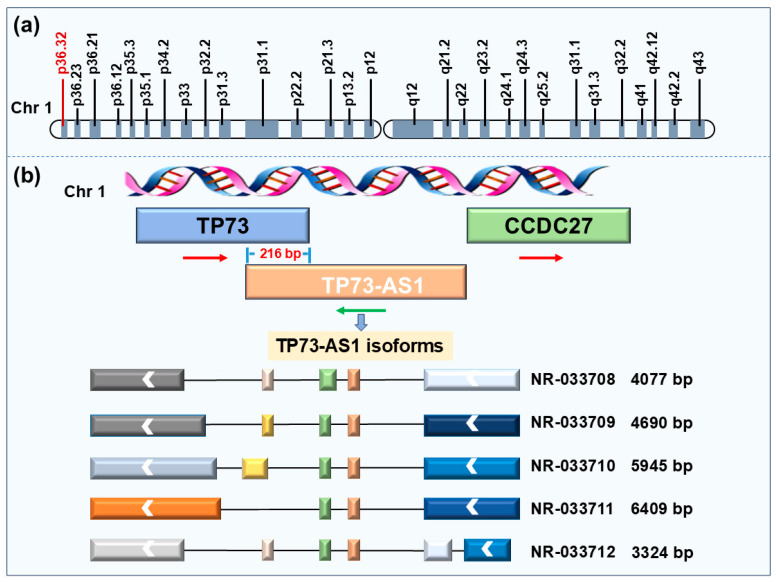
The location and isoforms of TP73-AS1. (**a**) The location of TP73-AS1 on chromosome 1; (**b**) different transcript isoforms of TP73-AS1.

Among these, TP73-AS1 is a typical tail-to-tail antisense lncRNA. In the human genome, this molecule is located on chromosome band 1p36.32, positioned between the TP73 and CCDC27 genes (Figure 1a). TP73-AS1 is transcribed from the antisense strand of the TP73 gene promoter, with its 3′ end overlapping the adjacent untranslated region of the TP73 gene by approximately 216 bp [19]. This lncRNA contains five exons and produces five distinct transcript isoforms (Figure 1b). Notably, although the TP73 and CCDC27 genes are highly conserved among mammals, orthologs of TP73-AS1 have only been identified in apes and Old World monkeys (OWMs), but not in mice, rats, cattle, or chickens [20]. This evolutionary feature indicates that the transcriptional expression of TP73-AS1 is species-specific and restricted to humans, apes, and OWMs. Despite being a relatively recent evolutionary addition among lncRNAs, TP73-AS1 is ubiquitously expressed at high levels across 63 human tissues [20]. Particularly noteworthy is that the expression level of TP73-AS1 is, on average, 50-fold higher than that of its neighboring genes TP73 and CCDC27, suggesting it may play important regulatory roles in cells.

### 2.2. Dual Role of TP73-AS1 in Cancer

TP73-AS1 plays a complex and context-dependent role in tumorigenesis, exhibiting both oncogenic and tumor-suppressive functions across different cancer types. In most malignancies, TP73-AS1 acts as an oncogene by promoting cell proliferation and inhibiting apoptosis [21]. A well-characterized example is its role in CC, where TP73-AS1 is significantly upregulated and contributes to tumor progression by promoting CCND2 expression through the inhibition of *miR-607* [15]. Similar oncogenic functions of TP73-AS1 have been observed in other cancers, including GC [10] and glioma [22], suggesting its widespread role as a tumor-promoting factor in diverse malignancies. However, emerging evidence reveals a contrasting tumor-suppressive role for TP73-AS1 in specific cancer types, such as bladder cancer and MM [23]. In bladder cancer, the overexpression of TP73-AS1 suppresses epithelial–mesenchymal transition (EMT) by downregulating MMP-2 and MMP-9 expression while upregulating E-cadherin, thereby inhibiting tumor invasion and metastasis [17]. Zhan et al. reported a significant downregulation of TP73-AS1 expression in MM, although the underlying molecular mechanisms remain to be fully elucidated [9]. In a complementary study, Wong et al. identified TP73-AS1 methylation in MM, providing compelling evidence that epigenetic modification may serve as a primary mechanism responsible for the observed TP73-AS1 suppression [24]. These findings highlight the duality of TP73-AS1 in cancer, which is regulated by the tissue-specific microenvironment. Further investigation of its regulatory network and mechanisms is essential for the development of context-specific therapeutic strategies targeting this lncRNA.

## 3. Regulatory Mechanisms of TP73-AS1 in Cancer

LncRNA is a key regulatory molecule in cells and exerts its functions through multiple mechanisms. At the transcriptional level, it can regulate the activity of transcription factors to influence gene transcription [25,26]. Antisense LncRNA binds to the sense-strand mRNA to regulate alternative splicing or form siRNA [27,28]. LncRNA can also bind to specific miRNAs through the “sponge effect” to regulate downstream genes [29,30] or act as a precursor of certain mRNAs. At the DNA level, it recruits chromatin modification factors to regulate the expression state of DNA and can also bind to specific proteins to participate in various biological functions. Together, these functions underscore the critical roles of lncRNAs in gene regulation and cellular processes. TP73-AS1, one of the LncRNAs, primarily exerts its oncogenic function by directly or indirectly regulating the expression of related genes through the following three aspects.

### 3.1. Binding to Proteins

**Figure 2:**
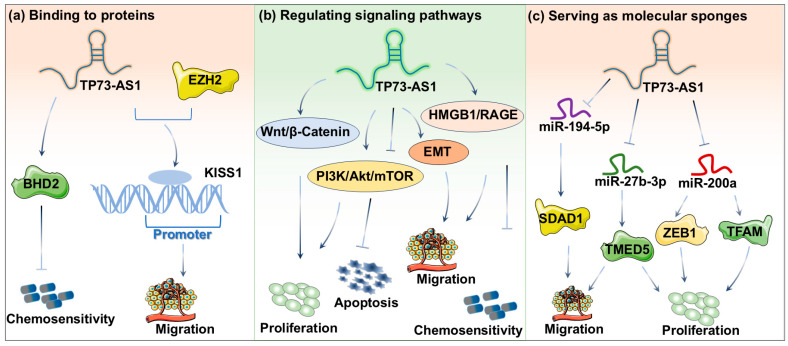
Different regulatory mechanisms of TP73-AS1 in cancer. (**a**) TP73-AS1 binds to different proteins and achieves certain biological functions; (**b**) TP73-AS1 affects its function by regulating various signaling pathways; (**c**) TP73-AS1 serves as a molecular sponge that binds to different microRNAs.

TP73-AS1 can regulate the stability and activity of proteins by binding to specific proteins (Figure 2a). KISS1, as a key metastasis suppressor, is closely related to tumor progression. The activation of KISS1 can effectively reduce the motility and invasive ability of tumor cells [31]. In clear-cell renal cell carcinoma, TP73-AS1 interacts with Enhancer of zest homolog 2 (EZH2) and then specifically binds to the promoter region of the KISS1 gene, thereby inhibiting the expression of KISS1 and ultimately promoting cell proliferation and migration [32]. Similarly, in esophageal cancer, knocking down the expression of TP73-AS1 in EC9706 and KYSE30 cells leads to a decrease in the expression of BHD2, thereby enhancing the drug sensitivity of esophageal cancer cells to 5-fluorouracil and cisplatin [33].

### 3.2. Regulating Signaling Pathways

TP73-AS1 can also function as an upstream regulatory factor in various cancer-related signaling pathways, modulating tumor cell proliferation, apoptosis, and chemosensitivity (Figure 2b). (1) Wnt/β-Catenin signaling pathway: in GC, the downregulation of TP73-AS1 destabilizes β-catenin and reduces its interaction with TCF-4, thereby suppressing the Wnt/β-catenin signaling pathway and ultimately inhibiting cell proliferation [11]. (2) PI3K/Akt/mTOR signaling pathway: The role of TP73-AS1 in the PI3K/Akt/mTOR pathway exhibits tissue-specific effects. In lung adenocarcinoma (LAD) tissues and cell lines, the PI3K/AKT signaling pathway is significantly activated, while silencing TP73-AS1 expression markedly suppresses its activity, suggesting that TP73-AS1 may promote LAD progression by activating the PI3K/AKT pathway [13]. In contrast, in clear-cell renal cell carcinoma, TP73-AS1 expression levels are negatively correlated with PI3K/Akt/mTOR signaling activity. Specifically, TP73-AS1 overexpression significantly reduces the ratios of p-AKT/AKT and p-mTOR/mTOR [32]. (3) HMGB1/RAGE signaling pathway: In HCC, TP73-AS1 upregulates the HMGB1/RAGE signaling axis, promoting the expression of pro-inflammatory cytokines (e.g., IL-6, IL-1β, and TNF-α), thereby significantly enhancing the malignant proliferation of HCC cells [16]. Similarly, in glioblastoma, TP73-AS1-mediated activation of the HMGB1/RAGE pathway enhances tumor cell proliferation and invasion, confirming its role as a critical oncogenic lncRNA [34]. However, in GC, knockdown of TP73-AS1 effectively suppresses GC cell proliferation and induces apoptosis by targeting the HMGB1/RAGE pathway, while also significantly increasing tumor cell sensitivity to cisplatin chemotherapy [35]. (4) EMT pathway: in GC, high expression of TP73-AS1 significantly enhanced the migration and invasion of GC cells by promoting the expression of N-cadherin and Snai, key effector molecules of the EMT pathway [36].

### 3.3. Serving as Molecular Sponges

In cancer, the primary mechanism of action of lncRNA is to sponge miRNAs, forming a lncRNA-miRNA-mRNA regulatory network. Like most lncRNAs, TP73-AS1 primarily functions as a competitive endogenous RNA (ceRNA), acting as an mRNA “sponge” to regulate downstream gene expression (Figure 2c). TP73-AS1 functions as an “endogenous sponge” by sequestering *miR-27b-3p*, thereby upregulating the expression of the transmembrane P24 trafficking protein 5 (TMED5). This mechanism effectively promotes the proliferation, migration, and invasive capabilities of GC cells [10]. Li et al. further demonstrated that TP73-AS1 competitively binds to *miR-194-5p*, enhancing SDAD1 expression and accelerating the metastatic progression of GC [37]. However, *miR-200a* exhibits a high expression profile in various tumor tissues and is recognized for its tumor-suppressive properties. Studies by Yao et al. revealed that TP73-AS1 competitively interacts with mitochondrial transcription factor A (TFAM) for *miR-200a* binding, which amplifies TFAM expression and subsequently drives the proliferation of breast cancer cells [38]. Additionally, TP73-AS1 promotes breast cancer cell proliferation and enhances mitogen activity by competitively binding to *miR-200a* at the *3′*-UTR region of zinc finger E-box-binding homeobox 1 (ZEB1), thereby upregulating ZEB1 expression [39]. Moreover, TP73-AS1 can also competitively bind to other microRNAs and affect malignant biological behaviors such as tumor proliferation, invasion, and metastasis by regulating downstream genes, for example, *miR-124* [22], *miR-142* [40], *miR-490-3p* [41], *miR-194* [14], and *miR-539-5p* [42]. Finally, we used the database starBase to search for miRNA that can interact with TP73-AS1, and the results are shown in Table 1. Some of the results are consistent with reports in the literature [43].

## 4. Functional Roles of TP73-AS1 in Cancer

### 4.1. Gastric Cancer

**Figure 3:**
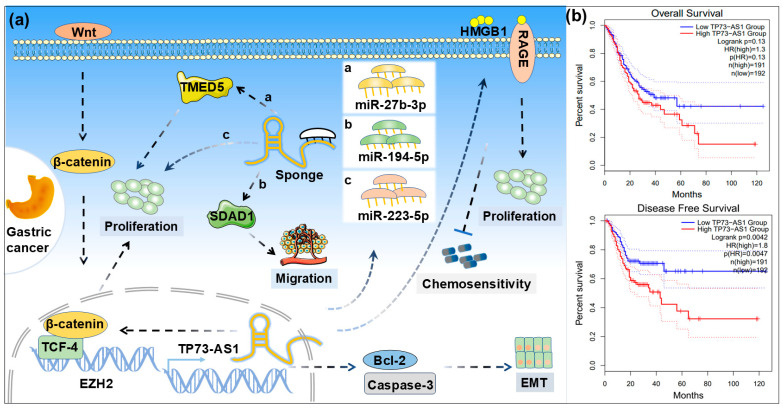
Roles and survival of TP73-AS1 in GC. (**a**) Schematic diagram of the regulatory mechanism of TP73-AS1 in GC (a: miR-27b-3p; b: miR-194-5p; c: miR-223-5p). (**b**) Kaplan–Meier plot of the effect of TP73-AS1 on overall survival and disease-free survival of GC (The dotted lines represent 95% confidence intervals for the corresponding group).

Gastric cancer is a common malignant tumor and one of the leading causes of cancer-related deaths worldwide [44]. Studies have shown that the expression level of the long non-coding RNA TP73-AS1 is significantly higher in GC tissues compared to normal tissues, and its expression level is closely associated with TNM stage, tumor invasion depth, lymph node metastasis, overall survival (OS), and prognosis in GC patients [10,11,45]. Bioinformatics analysis further confirmed that TP73-AS1 is one of the key molecules regulating Epstein–Barr virus-associated GC [46]. Research by Bao et al. revealed that TP73-AS1, as a direct target of *miR-27b-3p*, promotes the proliferation and invasion of GC cells by regulating the *miR-27b-3p*/TMED5 signaling axis [10]. Additionally, Wang et al. demonstrated that knockdown of TP73-AS1 significantly inhibits the proliferation, colony formation, and invasion capabilities of GC cells, while downregulating the expression levels of TCF-4 and β-catenin, suggesting that TP73-AS1 plays an important role in GC through the regulation of the Wnt/β-catenin signaling pathway [11]. TP73-AS1 has also been confirmed to function as a ceRNA, promoting the growth and metastasis of GC cells by regulating the *miR-194-5p*/SDAD1 axis [37]. The downregulation of TP73-AS1 promotes cell apoptosis by regulating the Bcl-2/caspase-3 pathway; inhibits cell proliferation, colony formation, and migration-invasion capabilities; and reverses the EMT process via its silencing, thereby suppressing the migration and invasion properties of GC cells [36]. Further research found that *miR-223-5p* promotes the invasion and migration of GC cells by inhibiting TP73-AS1 expression, while overexpression of TP73-AS1 produces the opposite effect, indicating that TP73-AS1 may serve as a potential prognostic marker for GC [47]. Notably, TP73-AS1 is closely related to chemotherapy resistance in GC. Research by Peng et al. showed that high expression of TP73-AS1 is associated with chemotherapy sensitivity in GC cells, and knockdown of TP73-AS1 enhances the sensitivity of GC cells to cisplatin and inhibits cell proliferation by regulating the HMGB1/RAGE signaling pathway [35]. Based on the above evidence, we constructed a schematic diagram of the regulatory mechanism of TP73-AS1 in GC (Figure 3a).

Further Kaplan–Meier survival analysis using databases indicated that while the OS of GC patients with high TP73-AS1 expression did not show significant statistical differences compared to those with low expression, there was a significant difference in disease-free survival (DFS) (Figure 3b). This finding suggests that TP73-AS1 may act as a key oncogenic factor in the development and progression of GC, providing a potential novel molecular target for individualized treatment and prognostic assessment in GC patients.

### 4.2. Lung Cancer

**Figure 4:**
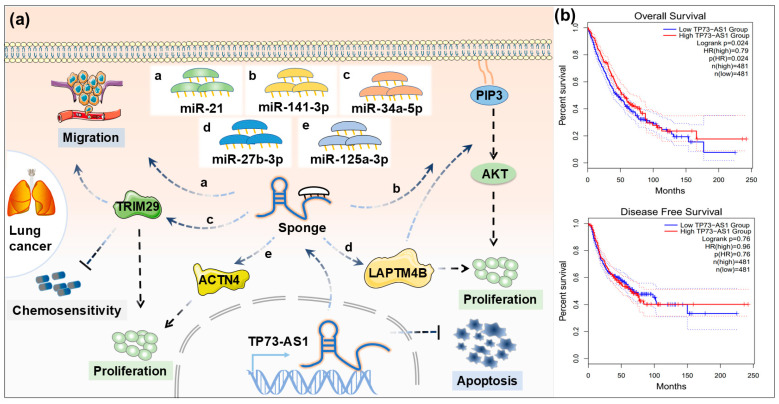
Roles and survival of TP73-AS1 in lung cancer. (**a**) Schematic diagram of the regulatory mechanism of TP73-AS1 in lung cancer (a: miR-21; b: miR-141-3p; c: miR-34a-5p; d: miR-27b-3p; e: miR-125a-3p). (**b**) Kaplan–Meier plot of the effect of TP73-AS1 on overall survival and disease-free survival of lung cancer (The dotted lines represent 95% confidence intervals for the corresponding group).

Lung cancer is the malignancy with the highest incidence and mortality rates worldwide [48]. Research by Zhu et al. found that the expression of TP73-AS1 was significantly elevated in tumor tissues of non-small cell lung cancer (NSCLC) patients compared to normal tissues, and its high expression was closely associated with poorer OS in patients [12]. TP73-AS1 promotes NSCLC progression through multiple molecular pathways: on one hand, TP73-AS1 enhances tumor cell invasion and migration by regulating the expression of *miR-21* [12] and promotes tumor cell proliferation by binding to *miR-141-3p* [49]; on the other hand, TP73-AS1 can also activate the PI3K/Akt signaling pathway to promote tumor proliferation [13]. Additionally, TP73-AS1 acts as a ceRNA to sponge *miR-34a-5p*, thereby relieving its inhibitory effect on the target gene TRIM29, ultimately promoting lung cancer cell proliferation, migration, invasion, cisplatin resistance, and inhibiting apoptosis [50]. TP73-AS1 can also inhibit the expression of *miR-27b-3p* by binding to it, which in turn regulates LAPTM4B and promotes lung cancer cell growth and metastasis [51]. Further research by Tong et al. demonstrated that knockdown of TP73-AS1 could inhibit the growth, migration, and invasion of NSCLC cells in vitro and suppress tumor growth in vivo. Through bioinformatics analysis and molecular mechanism studies, it was confirmed that TP73-AS1 interacts with *miR-125a-3p* to regulate the expression of ACTN4, thereby contributing to lung cancer progression [52]. Based on the above evidence, we developed a schematic diagram of the regulatory mechanism of TP73-AS1 expression in lung cancer (Figure 4a).

Kaplan–Meier survival analysis from the database revealed that lung cancer patients with high TP73-AS1 expression had significantly lower OS rates compared to those with low expression (*p* < 0.05), although no significant difference was observed in DFS between the two groups (Figure 4b). These findings indicate that TP73-AS1 plays a critical role in lung cancer progression, and its expression level is closely associated with patient prognosis, suggesting that TP73-AS1 may serve as a novel potential target for the diagnosis and treatment of lung cancer.

### 4.3. Colorectal Cancer

Colorectal cancer is the third most common malignant tumor worldwide and the second leading cause of cancer-related deaths. Recent studies have shown that the long non-coding RNA TP73-AS1 is significantly upregulated in CRC, and its overexpression is closely associated with distant metastasis and advanced clinical stages of the tumor. Experimental evidence indicates that knockdown of TP73-AS1 significantly inhibits the growth, proliferation, invasion, and migration capabilities of CRC cells [42,53]. At the molecular level, TP73-AS1 can bind to *miR-194* and negatively regulate its expression, thereby influencing CRC progression. Additionally, the expression of TP73-AS1 positively regulates the expression of transforming growth factor-α (TGF-α) [14]. Ding et al. further demonstrated that TP73-AS1 acts as a molecular sponge for *miR-539-5p*, upregulating the expression of secreted phosphoprotein 1 (SPP-1), thereby promoting the malignant progression of CRC, including enhanced cell proliferation, migration, and invasion [42]. Moreover, the expression of TP73-AS1 is positively correlated with the expression of transforming growth factor-β1 (TGF-β1) [54]. Notably, the overexpression of TP73-AS1 not only significantly inhibits the growth of CRC cells but also promotes apoptosis, characterized by the downregulation of Bcl-2 levels and increased expression of caspase-3. Specifically, TP73-AS1 regulates PTEN expression by acting as a ceRNA for *miR-103*, thereby suppressing CRC cell proliferation [55]. In summary, TP73-AS1 may serve as an important oncogene in CRC, with potential value as a diagnostic marker and therapeutic target. However, the specific mechanisms of TP73-AS1 and its functions in the tumor microenvironment require further in-depth investigation.

### 4.4. Cervical Cancer

Cervical cancer is one of the malignant tumors that poses a serious threat to women′s lives and health. A study found that TP73-AS1 was upregulated in CC tissues and was associated with lower survival in CC patients [56]. Zhang et al. collected and evaluated CC and adjacent tissues from 56 patients and found that TP73-AS1 levels were upregulated in CC tissues and the OS rate was poor. Furthermore, *miR-607* was found to be negatively regulated by TP73-AS1, while CCND2 was negatively regulated by *miR-607*, indicating that upregulation of TP73-AS1 promoted CCND2 by inhibiting *miR-607* expression and thus promoted CC progression [15]. Xu et al. found that TP73-AS1 regulates CC progression by competitively binding to *miR-329-3p*, which in turn regulates cervical cell proliferation and migration [57]. Another study showed that decreased expression of TP73-AS1 reduced tumor size and downregulated SMAD2 gene expression, suggesting that it regulates SMAD2 gene expression by targeting *miR-329-3p* to promote CC cell proliferation [58]. Therefore, targeting TP73-AS1 may be a novel lncRNA-based strategy to improve the treatment and prognosis of CC.

### 4.5. Hepatocellular Carcinoma

Hepatocellular carcinoma is a common gastrointestinal tumor, ranking fifth in incidence of malignant tumors and third in mortality [59]. Li et al. proposed that TP73-AS1 can bind to *miR-200a*, and since *miR-200a* is a receptor for high-mobility group protein B1 (HMGB1), the expression of TP73-AS1 is positively correlated with HMGB1. TP73-AS1 promotes the proliferation of HCC cells by regulating the HMGB1/RAGE signaling pathway [16]. Additionally, Chen et al. found that in HCC, TP73-AS1 can negatively regulate the expression of *miR-539*. The knockdown of TP73-AS1 or overexpression of *miR-539* can inhibit the growth of HCC and reduce the infiltration of M2-type macrophages in vivo [60]. Ma et al. further pointed out that the expression of TP73-AS1 in HCC is negatively correlated with *miR-103*, although its specific mechanism of action still requires further clarification [61]. In summary, these findings not only deepen our understanding of the role of TP73-AS1 in the mechanisms of HCC but also provide a theoretical basis for the future development of targeted therapeutic strategies against TP73-AS1 and its related molecular networks.

### 4.6. Medulloblastoma

Medulloblastoma (MB) is the most common malignant brain tumor in children, and its incidence has been increasing annually [62]. Varon et al. analyzed the expression levels of TP73-AS1 in 216 normal tissue samples and 552 MB samples, revealing that TP73-AS1 is significantly upregulated in MB tissues. Further experiments demonstrated that high expression of TP73-AS1 promotes tumor cell proliferation and migration, enhances cell viability, and inhibits apoptosis. Conversely, the knockdown of TP73-AS1 produces the opposite effect, significantly suppressing tumor growth and prolonging the survival time of tumor-bearing mice [63]. Additionally, studies by Li et al. further confirmed the oncogenic role of TP73-AS1, showing that TP73-AS1 acts as a sponge for *miR-494-3p*, positively regulating the expression of EIF5A2, thereby promoting the progression of MB [64]. These discoveries not only deepen our understanding of the pathogenesis of MB but also provide a crucial theoretical foundation for the future development of TP73-AS1-targeted therapeutic strategies.

### 4.7. Other Cancers

TP73-AS1 also showed various degrees of aberrant expression in other cancers. Most of them showed elevated expression, which promoted cancer cell proliferation and contributed to cancer development. The mechanisms were similar, generally through serving as a ceRNA combined with different miRNAs, thus regulating downstream genes and signaling pathways, such as in breast cancer [38], osteosarcoma [65], prostate cancer [66], glioma [22,34], and pancreatic cancer [67]. Interestingly, it was found that TP73-AS1 also functions as a tumor suppressor gene in bladder cancer [17] and MM [24]. Finally, we summarize the role of TP73-AS1 in various human cancers and the mechanisms involved (Table 2), and its importance extends to its potential to be a breakthrough target for cancer diagnosis and treatment strategies.

## 5. Clinical Applications of TP73-AS1 in Cancer

Despite significant progress in cancer treatment in recent years, numerous challenges remain to be addressed, such as chemoresistance, tumor metastasis, cancer recurrence, and diagnostic delays, all of which contribute to poor patient prognosis [69]. LncRNAs have emerged as promising clinical biomarkers for cancer diagnosis, progression monitoring, recurrence assessment, and prognosis prediction [70,71,72]. Among them, lncRNA TP73-AS1 exhibits differential expression across multiple cancers, and its SNPs are significantly associated with cancer susceptibility. These findings underscore its potential clinical utility in both diagnostic and therapeutic applications.

### 5.1. As a Diagnostic Marker

Early diagnosis is crucial for improving the survival rates of cancer patients; however, it faces significant challenges due to the lack of obvious symptoms or the presence of only mild, non-specific signs [73]. The stable presence of the lncRNA TP73-AS1 in body fluids (such as blood and urine) makes it a promising novel non-invasive diagnostic biomarker for cancer patients.

Studies have shown that TP73-AS1 is significantly upregulated in various gastrointestinal tumors, including esophageal cancer (EC), HCC, GC, and CC, and it is closely associated with reduced patient survival rates, suggesting its potential as an auxiliary diagnostic biomarker for gastrointestinal tumors [74]. Analysis of the TCGA database revealed that high expression of TP73-AS1 is significantly associated with poor prognosis in CESC patients, and diagnostic models constructed by combining it with other miRNAs (such as *miR-128-3p* and *miR-142-3p*) can significantly improve diagnostic accuracy [75]. Furthermore, a study quantified the expression levels of 14 lncRNAs (such as HAGLR and TP73-AS1) in cancerous and normal tissues from 92 non-small cell lung cancer (NSCLC) patients using qPCR and constructed a predictive model using machine learning algorithms. The results demonstrated that these lncRNAs effectively distinguished cancerous tissues from normal tissues (AUC = 0.98) and differentiated NSCLC subtypes (AUC = 0.84), suggesting that lncRNAs like TP73-AS1 could serve as potential auxiliary tools for the early detection and histological diagnosis of NSCLC [76]. In addition, TP73-AS1 polymorphisms and expression levels are implicated in cancer susceptibility and progression, also supporting its potential as a diagnostic biomarker. In GC, the G allele of the TP73-AS1 rs3737589 locus significantly increased GC risk, and the variant genotypes (AG+GG) were associated with deeper tumor invasion (T3+T4 stage) [45]. In CRC, high expression of TP73-AS1 is associated with an advanced TNM stage and worse prognosis. Among the polymorphisms studied, rs9800 C>G was linked to a higher risk of CRC, while rs1181866 C>A was associated with a reduced risk of CRC [53]. Notably, a subsequent study by Gao et al. [68] further revealed that while the rs3737589 polymorphism was not associated with CRC susceptibility, it showed a significant correlation with the disease stage. The expression of TP73-AS1 in CRC tissues was lower in the CC genotype than in the TT genotype, and patients carrying the C allele had a reduced risk of developing stage III/IV tumors.

**Figure 5:**
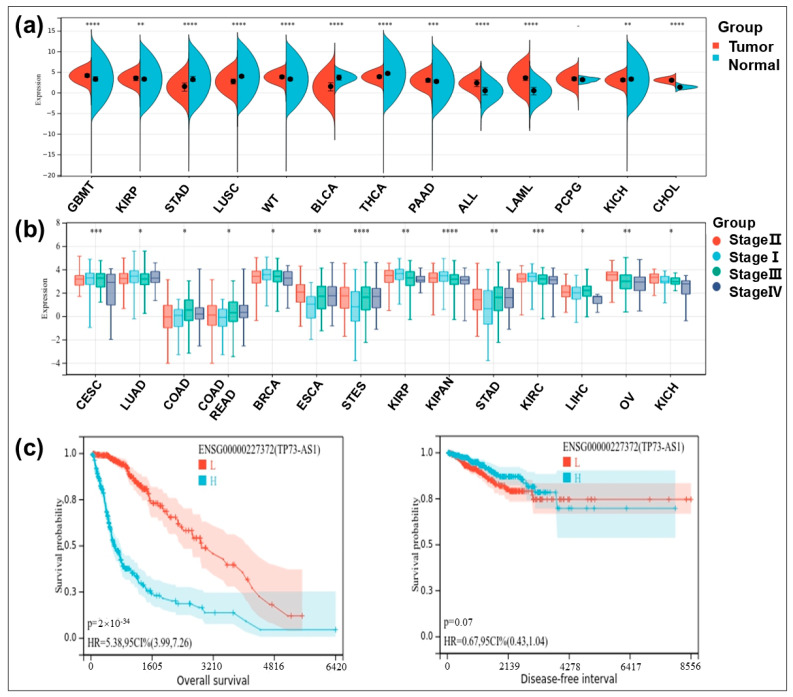
Results of bioinformatics analysis of TP73-AS1. (**a**) The differential expression of TP73-AS1 in different cancers and adjacent noncancerous tissues. (**b**) The differential expression of TP73-AS1 in different cancer stages. (**c**) Kaplan–Meier plot of the effect of TP73-AS1 on overall survival and disease-free survival of cancer patients. * *p* < 0.05; ** *p* < 0.01; *** *p* < 0.001; **** *p* < 0.0001.

To further validate TP73-AS1 as a clinical diagnostic or prognostic biomarker across multiple cancers, we first evaluated differences in TP73-AS1 expression between normal and tumor samples using unpaired Wilcoxon rank-sum and signed-rank tests. The results demonstrated that TP73-AS1 exhibited significantly elevated expression levels in glioblastoma (GBM) and cholangiocarcinoma (CHOL) (*p* < 0.001). Conversely, in bladder urothelial carcinoma (BLCA), TP73-AS1 showed markedly reduced expression (*p* < 0.001), which is consistent with previous studies (Figure 5a). Subsequently, we analyzed TP73-AS1 expression across different clinical stages of tumors using the Sangerbox database (http://www.sangerbox.com/tool, accessed on 15 July 2024). Unpaired Student’s *T*-tests and ANOVA were applied to assess pairwise and multi-group differences, respectively. Significant differences in TP73-AS1 expression were observed across clinical stages of GC and esophageal carcinoma (Figure 5b). Finally, cancer patients were stratified into two groups based on TP73-AS1 expression (50%). Log-rank tests revealed that TP73-AS1 expression was significantly associated with OS (*p* < 0.05) but not with DFS (Figure 5c).

### 5.2. As a Therapeutic Target

Due to its close association with tumorigenesis and drug resistance, TP73-AS1 has emerged as a significant potential therapeutic target in oncology. Extensive preclinical studies have demonstrated that suppressing TP73-AS1 expression through RNA interference technology significantly inhibits tumor cell proliferation and migration while inducing apoptosis in various tumor cell lines and mouse models [11,33,40]. Currently, inhibition strategies for TP73-AS1 primarily focus on small interfering RNA (siRNA), while research on antisense oligonucleotides (ASOs) remains relatively limited, offering potential directions for exploring novel targeted therapies. In addition, in terms of drug resistance, Peng et al. found that the overexpression of TP73-AS1 is associated with enhanced drug resistance in GC cells, and reducing the expression of TP73-AS1 can enhance the sensitivity of GC cells to the chemotherapeutic drug cisplatin [35]. Similarly, in glioblastoma, high expression of TP73-AS1 is not only closely linked to poor prognosis but also increases the resistance of glioblastoma stem cells to temozolomide [77]. Based on existing research, the biological functions and clinical significance of TP73-AS1 in various malignancies have been well-established. Future studies should focus on developing more efficient TP73-AS1-targeted inhibition strategies and elucidating its molecular mechanisms in tumor drug resistance, thereby providing new theoretical foundations and therapeutic approaches for cancer treatment.

## 6. Conclusions and Future Perspectives

LncRNA serves as a crucial regulatory factor in tumor formation and development, and its impact on cancer progression is attracting increasing attention. TP73-AS1 exhibits tissue-specific expression patterns and plays differential regulatory roles in various malignancies. Studies have shown that this gene is significantly upregulated in tumor tissues such as GC, lung cancer, and CC, where it drives tumor progression by promoting cell proliferation, migration, and invasion. However, in bladder cancer and multiple myeloma, TP73-AS1 displays low expression characteristics and functions as a tumor suppressor (Table 2). This unique dual role makes TP73-AS1 a highly promising therapeutic target: for tumors with high expression (e.g., GC, lung cancer), its pro-oncogenic activity can be inhibited using gene-silencing technologies, whereas for tumors with low expression (e.g., bladder cancer), restoring its expression may reactivate its tumor-suppressive function, thereby enabling precise personalized treatment. At the molecular level, TP73-AS1 contributes to tumorigenesis and progression by modulating multiple critical signaling pathways, including Wnt/β-catenin, PI3K/AKT/mTOR, and HMGB1/RAGE. Building on this mechanism, combining TP73-AS1-targeted therapy with pathway-specific inhibitors (such as AKT inhibitors or Wnt pathway blockers) could produce synergistic effects, more effectively disrupting pro-oncogenic signaling. This strategy holds promises for enhancing therapeutic efficacy while reducing the risk of drug resistance. Furthermore, three SNPs in the TP73-AS1 gene (rs3737589, rs9800, and rs1181866) show significant associations with the risk of GC and CC. These genetic variants may serve as potential molecular biomarkers for identifying high-risk populations through genetic screening. When combined with TP73-AS1 expression profiling, this approach could provide novel molecular insights for early cancer diagnosis and personalized risk assessment.

At present, research on TP73-AS1 is still in its infancy, much like only the tip of the iceberg is visible. Although the majority of studies have recognized its clinical potential, there remains a considerable distance to its practical clinical application. First and foremost, delving into its specific regulatory mechanisms is of utmost urgency. In particular, it is essential to explore the interaction relationships between TP73-AS1, miRNA, and downstream target genes; expand the scope of research on the impact of other signaling pathways; and deepen our understanding of its regulatory network in the pathogenesis of tumors. Secondly, while the majority of research regards TP73-AS1 as an oncogene, some papers indicate that in a few types of cancer, TP73-AS1 acts as a tumor suppressor. This functional heterogeneity suggests that integrating high-depth, targeted lncRNA single-cell RNA sequencing (scRNA-seq) data for cross-cancer analysis will help systematically elucidate the context-specific regulatory mechanisms of TP73-AS1 in different tumor microenvironments and uncover its potential dual regulatory roles during cancer progression. Finally, target selection holds a pivotal position in drug development. Thus, exploring small-molecule drugs that can target TP73-AS1, conducting gene therapy research, and integrating the regulation of TP73-AS1 with existing treatment methods such as chemotherapy and radiotherapy are expected to overcome the problem of tumor drug resistance and become potential treatment directions and strategies for malignant tumors. Consequently, through in-depth exploration of the aforementioned research directions, we will be able to gain a more comprehensive understanding of the complex role of TP73-AS1 in cancer. This will provide a theoretical foundation for optimizing cancer diagnosis, prognostic evaluation, and treatment strategies, ultimately improving clinical outcomes for patients.

## Figures and Tables

**Table 1:** The miRNA interacting with TP73-AS1 was obtained through starBase database.

Name	MirAccession	TargetSites	BioComplex	ClipReadNum
hsa-miR-200a-3p	MIMAT0000682	2	4	27
hsa-miR-197-3p	MIMAT0000227	1	2	0
hsa-miR-488-3p	MIMAT0004763	1	1	15
hsa-miR-194-5p	MIMAT0000460	1	2	0
hsa-miR-107	MIMAT0000104	1	1	5
hsa-miR-326	MIMAT0000756	1	2	0
hsa-miR-141-3p	MIMAT0000432	2	4	27
hsa-miR-329-3p	MIMAT0001629	1	4	28
hsa-miR-494-3p	MIMAT0002816	1	1	3
hsa-miR-539-5p	MIMAT0003163	1	2	40
hsa-miR-485-5p	MIMAT0002175	1	1	12
hsa-miR-154-5p	MIMAT0000452	2	2	0
hsa-miR-193b-3p	MIMAT0002819	1	2	0
hsa-miR-193a-3p	MIMAT0000459	1	2	0
hsa-miR-27a-3p	MIMAT0000084	1	5	24
hsa-miR-330-5p	MIMAT0004693	1	2	0
hsa-miR-125a-3p	MIMAT0004602	1	2	0
hsa-miR-128-3p	MIMAT0000424	1	5	24
hsa-miR-103a-3p	MIMAT0000101	1	1	5
hsa-miR-124-3p	MIMAT0000422	1	2	0

**Table 2:** Clinical characteristics of TP73-AS1 in different cancers.

Cancer Types	Expression	Related Gene	Function	Gene Type	Reference
Gastric cancer	↑	*MiR-27b-3p*/TMED5	Proliferation	Oncogene	[10]
		TCF-4/β-catenin	Proliferation, migration		[11]
		HMGB1/RAGE	Chemosensitivity		[35]
		Bcl-2/caspase-3	Apoptosis, EMT		[36]
		*MiR-194-5p*/SDAD1	Migration		[37]
		*MiR-223-5p*	Proliferation, invasion		[47]
Lung cancer	↑	*MiR-21*	Migration, invasion	Oncogene	[12]
		PI3K/Akt	Proliferation		[13]
		*MiR-141-3p*	Proliferation		[49]
		*MiR-34a-5p*/TRIM29	Proliferation, migration Chemosensitivity		[50]
		*MiR-27b-3p*/LAPTM4B	Proliferation, migration		[51]
		*MiR-125a-3p*/ACTN4	Proliferation, invasion		[52]
Colorectal cancer	↑	*MiR-194*/TGF-α	Proliferation	Oncogene	[14]
		*MiR-539-5p*/SPP-1	Proliferation, migration		[42]
		TGF-β1	Proliferation, migration		[54]
		*MiR-103*/PTEN	Apoptosis		[55]
Cervical cancer	↑	*MiR-607*/CCND2	Proliferation	Oncogene	[15]
		/	Proliferation, migration		[56]
		*MiR-329-3p*/ARF1	Proliferation, migration		[57]
		*MiR-329-3p*/SMAD2	Proliferation		[58]
Hepatocellular carcinoma	↑	*MiR-200a*/HMGB1/RAGE	Proliferation	Oncogene	[16]
		*MiR-539*	Proliferation		[60]
		*MiR-103*	Apoptosis		[61]
Glioma	↑	*MiR-124*/iASPP	Proliferation, migration	Oncogene	[22]
		*MiR-142*/HMGB1/RAGE	Proliferation, invasion		[34]
		ALDH1A1	Chemotherapy resistance		[68]
Clear-cell renal cell carcinoma	↑	EZH2/KISS1	Proliferation, migration	Oncogene	[32]
Esophageal cancer	↑	BDH2/caspase-3	Chemosensitivity	Oncogene	[33]
Breast cancer	↑	*MiR-200a*/TFAM	Proliferation	Oncogene	[38]
		*MiR-200a*/ZEB1	Migration, invasion		[39]
		*MiR-490-3p*/TWIST1	Vasculogenic mimicry		[41]
Osteosarcoma	↑	*MiR-142*/Rac1	Migration	Oncogene	[40]
		/	Migration, invasion		[65]
Medulloblastoma	↑	/	Proliferation, migration	Oncogene	[63]
		*MiR-494-3p*/EIF5A2	Proliferation		[64]
Prostate cancer	↑	*MiR-320a*	Migration	Oncogene	[66]
Pancreatic cancer	↑	*MiR-128-3p*/GOLM1	Migration	Oncogene	[67]
Bladder cancer	↓	MMP-2/MMP-9	Migration, invasion	Anti- oncogene	[17]
Multiple myeloma	↓	/	/	Anti- oncogene	[9,24]

↑: Upregulation; ↓: Downregulation.

## Supplementary Materials

The supporting information can be downloaded at: https://www.mdpi.com/article/10.3390/ijms26083886/s1.
