# Supplementary material for: Long Non-Coding TP73-AS1: A Potential Biomarker and Therapeutic Target in Cancer
Source: Int J Mol Sci. 2025 Apr 20;26(8):3886. doi: 10.3390/ijms26083886 (PMC12028249; doi:10.3390/ijms26083886)
Supplement: Supplementary file 1 [file ijms-26-03886-s001.zip › ijms-3526526-supplementary.pdf]

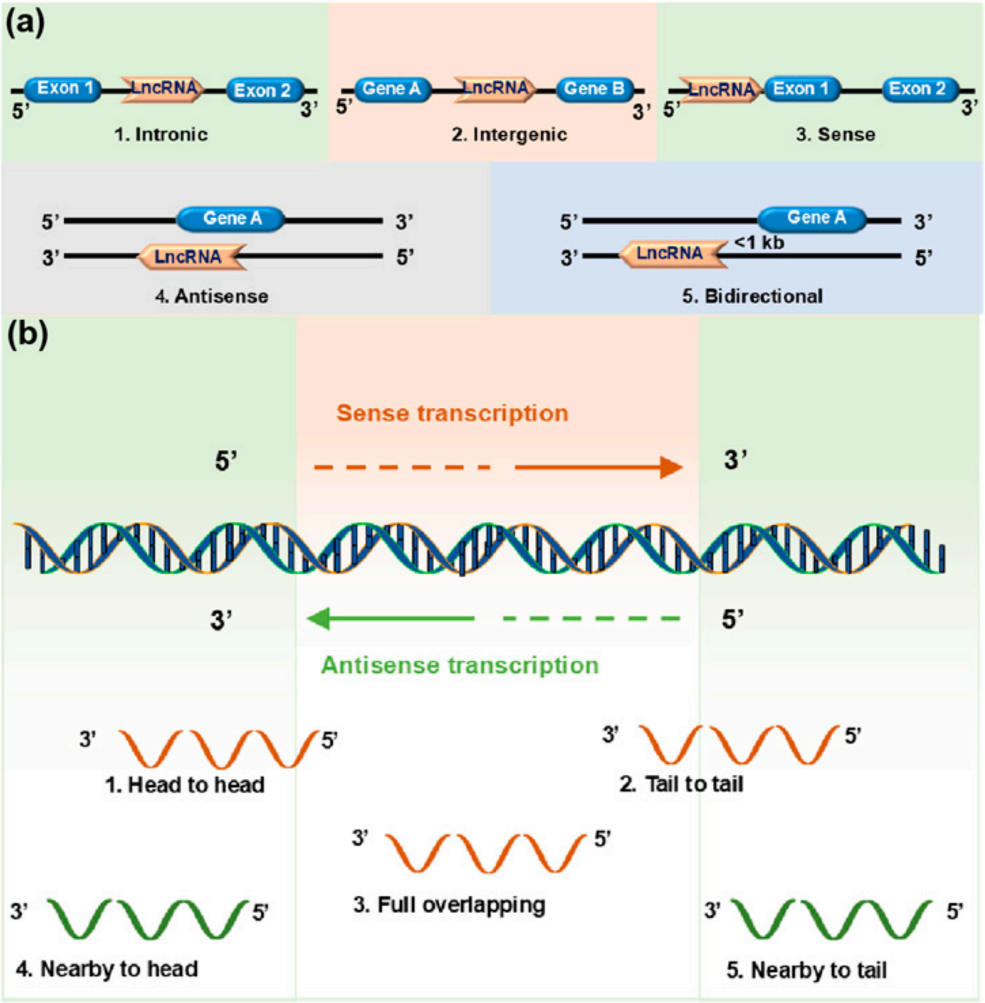

Figure S1. Classification of lncRNAs. (**a**) Schematic representation of different IncRNAs location; (**b**) Types of lncRNAs on antisense strands.
